# Recent Advances in the HPPH-Based Third-Generation Photodynamic Agents in Biomedical Applications

**DOI:** 10.3390/ijms242417404

**Published:** 2023-12-12

**Authors:** Lixiao Fan, Zheng Jiang, Yu Xiong, Zepeng Xu, Xin Yang, Deying Gu, Mailudan Ainiwaer, Leyu Li, Jun Liu, Fei Chen

**Affiliations:** 1 Department of Otolaryngology-Head and Neck Surgery, West China Hospital, Sichuan University, Chengdu 610064, China; fanlixiao2017@163.com (L.F.); drjiangzhengmd@gmail.com (Z.J.); tracy_xiongyu@163.com (Y.X.); 18010676430@163.com (X.Y.); ebhkgdy@163.com (D.G.); mawluda226@163.com (M.A.); 15228208034@163.com (L.L.); 2 Head and Neck Surgical Center, West China Hospital, Sichuan University, Chengdu 610064, China; 3 West China Clinical Medical College, Sichuan University, Chengdu 610064, China; m15002093314@163.com

**Keywords:** photodynamic, HPPH, third generation, photosensitizer

## Abstract

Photodynamic therapy has emerged as a recognized anti-tumor treatment involving three fundamental elements: photosensitizers, light, and reactive oxygen species. Enhancing the effectiveness of photosensitizers remains the primary avenue for improving the biological therapeutic outcomes of PDT. Through three generations of development, HPPH is a 2-(1-hexyloxyethyl)-2-devinyl derivative of pyropheophorbide-α, representing a second-generation photosensitizer already undergoing clinical trials for various tumors. The evolution toward third-generation photosensitizers based on HPPH involves structural modifications for multimodal applications and the combination of multifunctional compounds, leading to improved imaging localization and superior anti-tumor effects. While research into third-generation HPPH is beneficial for advancing PDT treatment, equal attention should also be directed toward the other two essential elements and personalized diagnosis and treatment methodologies.

## 1. Introduction

Cancer stands as one of the most devastating diseases globally. Clinical treatments primarily involve surgery, radiation therapy, and chemotherapy. However, their overall effectiveness is often limited due to the invasive nature of surgery, the unpredictable toxicity of radiation and chemotherapy, drug resistance, and long-term adverse reactions. These factors lead to a lower quality of life for cancer patients, a higher risk of recurrence and mortality, making clinical cures challenging to achieve. Photodynamic therapy (PDT) is emerging as a novel, gradually acknowledged non-invasive treatment method [1].

Records dating back over 3000 years from ancient Egypt, India, and China document the use of light in treating various skin diseases [2]. Over a century ago, the German medical student O. Raab first reported cell death caused by the interaction of light with chemicals. Due to the observed fluorescence emission during this process, he correlated the cytotoxic effect with the resulting fluorescence [3]. Concurrently, H. von Tappeiner and A. Jodlbauer discovered the pivotal role of oxygen in this process and coined the term “photodynamic action” to describe this phenomenon [4]. It was not until 1960 when R. Lipson and S. Schwartz discovered the diagnostic and therapeutic effects of a hematoporphyrin derivative (HpD) on cancer, jointly proposing the concept of PDT.

PDT comprises three fundamental elements: a photosensitizer (PS), specific wavelengths of light, and molecular oxygen. Their dynamic interaction selectively destructs target tissues. The PS itself does not directly interact with biological molecules. Instead, during light exposure, the PS absorbs light, acting as a catalyst to transfer energy from light to oxygen molecules, generating reactive oxygen species (ROS). The main components of ROS include singlet oxygen (^1^O_2_), superoxide radicals (O_2_^−•^), hydroxyl radicals (HO^•^), and hydrogen peroxide (H_2_O_2_) [5]. Substantial ROS generation initiates a cascade of biochemical events in cells, inducing damage and death in target tissues.

**Figure 1:**
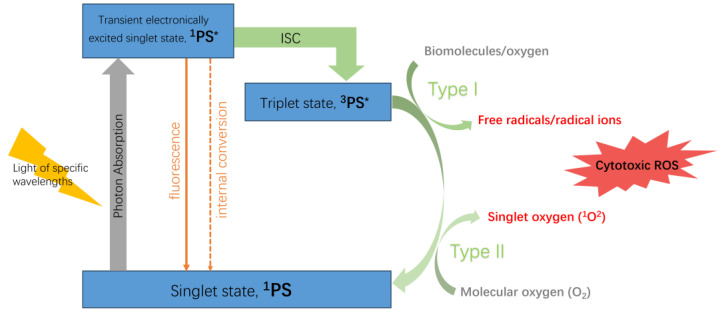
Mechanism of PDT.

The photodynamic reaction occurs in two types: Type I and Type II, as shown in Figure 1. When a photosensitizer (PS) absorbs light of specific wavelengths, it transitions from the ground state (singlet state, ^1^PS) to a transient electronically excited singlet state (^1^PS*), a very brief process that often lasts only a few nanoseconds or even shorter [6]. However, the ^1^PS* state is highly unstable and can dissipate excess energy through emission of light (fluorescence) or heat (internal conversion), returning to the ground state. Nonetheless, the singlet state can also undergo intersystem crossing (ISC) between systems, transitioning through the spin flipping of electrons in high-energy orbitals to a more stable, long-lived electronically excited state (triplet state, ^3^PS*).

In the Type I reaction, the ^3^PS* directly reacts with substrates (biomolecules or oxygen), causing hydrogen atom abstraction or electron transfer, generating various free radicals or radical ions. These produced radicals further react with molecular oxygen to generate ROS, inducing cellular damage. The ^3^PS* state, more stable and capable of existing for tens of microseconds, has sufficient time to directly transfer energy to molecular oxygen (O_2_), leading to the formation of singlet oxygen (^1^O_2_) while returning to the ground state. This process is termed the Type II reaction. The ^1^O_2_ generated from the Type II reaction interacts with biological molecules, inducing oxidative damage and ultimately leading to cell death. During PDT, the Type II reaction predominates, yet both Type I and Type II reactions can occur simultaneously [7]. Among the products, ^1^O_2_ serves as the primary cytotoxic ROS responsible for biological effects.

PDT achieves its therapeutic goals mainly through three pathways: inducing apoptosis, causing changes in blood dynamics or vascular disruption, and triggering local inflammatory responses that activate the immune system [8]. The overall effectiveness of PDT in treating tumors depends on various factors, including the type of PS, the dosage of PS and light exposure, the oxygen content and availability in the tumor microenvironment, and the interval between PS administration and light exposure [9].

Compared to traditional cancer treatments, PDT offers simplicity, minimal invasiveness, dual selectivity within the treatment area (localized light exposure or local PS accumulation), multiple comprehensive anti-tumor mechanisms, repeatability, and fewer systemic or long-term side effects. However, like any treatment, PDT has limitations. Localized irradiation has limited effect on metastatic lesions, and the depth of light penetration restricts the efficacy of treating deep tissue tumors. Additionally, the damaging effect on blood vessels leads to decreased oxygen levels in and around the tumor, impacting treatment efficacy [10].

The PS stands as the key factor in the biological efficacy of PDT. The primary approach to strengthening PDT effectiveness lies in designing and developing efficient PS, and often, enhancing tissue oxygen content by adding functional conjugates or co-carried materials [11]. The basic structures of PS include porphyrins, chlorins, anthocyanins, and other dyes like methylene blue, toluidine blue, rose bengal, and hypericin, categorized historically into first, second, and third generations [12]. The first generation, such as hematoporphyrin and its derivatives, notably photofrin, were the initial PS approved for tumor PDT treatment. However, they had drawbacks like lower chemical purity and shorter excitation wavelengths. Moreover, the heightened nonspecific phototoxicity stemmed from low selectivity and extended half-life [13]. Most second-generation PSs are based on the structures of porphyrin and chlorin, with improvements in purity, longer wavelength absorption, photosensitivity, and tissue selectivity. The third generation builds upon the second generation, often displaying superior performance. This involves structural conjugation with targeting capabilities specific to tumors or the tumor microenvironment [14]. Additionally, encapsulating PS into carriers like liposomes, micelles, or nanoparticles enhances the accumulation of PS at tumor sites [15]. Combining PS with chemotherapeutic or immunotherapeutic agents through various methods forms multi-mechanistic anti-tumor components, achieving targeted combination therapy against tumors.

**Figure 2:**
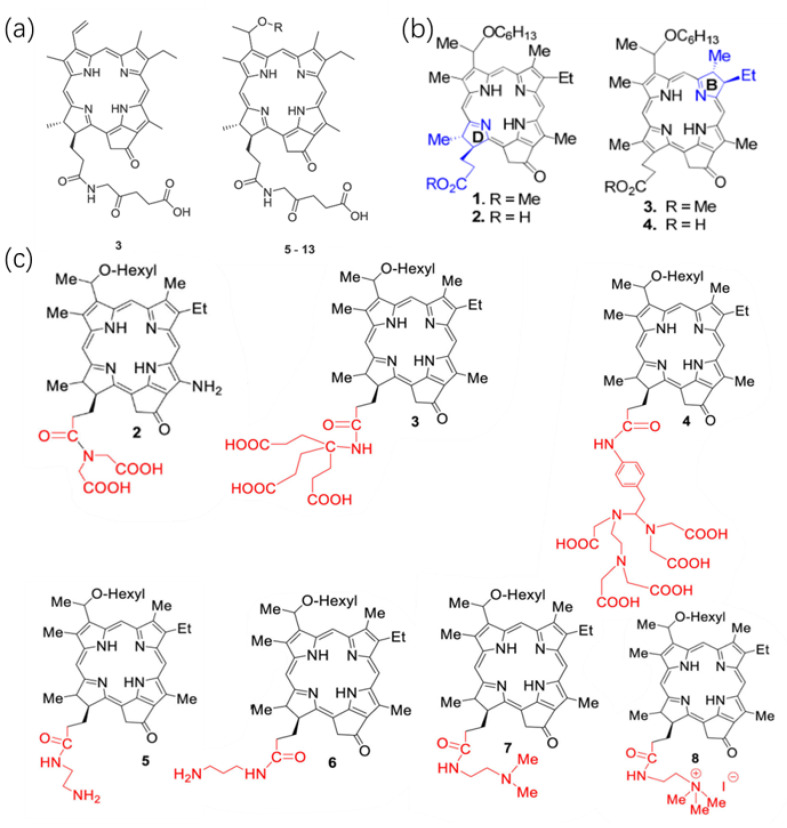
Direct Modification of The Innate Structure of HPPH. (**a**) Pyropheophorbide-a links with 5-ALA to produce compound **3**, compound **3** coupled with different chemical groups to synthesize compounds **5**–**13**, Rs are methyloxyethyl, ethyloxyethyl, n-propyloxyethyl/-butyloxyethyl/-pentyloxyethyl/-hexyloxyethyl/-ethoxyloxyethyl/-methoxyethyloxyethyl/-propoxyoxyethyloxyethyl. Reproduced with permission from ref. [20]. Copyright 2020, Elsevier. (**b**) Structure of HPPH **2** and corresponding chemically reduced ring ‘B’ isomer (iso-HPPH) **4**, HPPH methyl ester **1** and the corresponding ring-B reduced isomer 3. Reproduced with permission from ref. [21]. Copyright 2017, America Chemistry Society. (**c**) The HPPH analogs with variable carboxylic acid functionality **2**, **3**, **4**, alkyl amines **5**, **6**, N, N-dimethyl analog **7** and the quaternary ammonium salt **8**. Reproduced with permission from ref. [22]. Copyright 2022, Elsevier.

2-[1-Hexyloxyethyl]-2-devinyl pyropheophorbide-a (HPPH) is categorized as a second-generation photosensitizer, a 2-(1-hexyloxyethyl)-2-devinyl derivative of pyropheophorbide-a(PPa), as shown in Figure 2b. PPa was discovered by Hashimoto in 1962 in the abalone pancreas [16], derived from algal chlorophyll after the algae had been swallowed and metabolized. HPPH boasts excellent photophysical properties, higher accumulation in tumors, and mild skin photosensitivity. Its relatively high quantum yield also makes it a suitable fluorophore for optical fluorescence imaging. Therefore, HPPH holds promise as a dual-function agent for imaging and therapy, aiding in better treatment guidance by enhancing tumor visibility [17]. HPPH exhibits high lipophilicity and demonstrates slow plasma clearance, with nearly 100% of HPPH binding to diverse human plasma proteins and exhibiting no metabolic transformations [18]. Chen et al. constructed a physiologically based pharmacokinetic (PBPK) model for HPPH in rats, proposing the liver as the primary elimination organ [19]. Clinical trials using HPPH as a photosensitizer for PDT have been conducted in various cancer types, including bladder, colon, oral, basal cell carcinoma, salivary gland, nasopharyngeal, hypopharyngeal, laryngeal, esophageal, gastric, lung, thyroid, cervical cancer, and osteosarcoma.

However, using free HPPH alone for treatment presents three significant limitations: (1) Extensive diffusion and the non-specific uptake of HPPH diminish its effectiveness and lead to off-target effects; the mechanism of free HPPH primarily induces ROS to cause cell death of whichever cell uptakes it, indicating that it cannot entirely target cancer cells without affecting normal tissue. (2) If an insufficient immune response is elicited, it cannot stimulate systemic immune responses against tumor metastasis; thus, the treatment effect of HPPH remains localized [23]. (3) The intratumoral uptake and retention still remain tumor-specific, and some tumor types with low retention of HPPH have not shown ideal PDT efficacy [24].

In response to these limitations, research efforts have focused on modifying the physical structure of HPPH, loading it into new drug delivery systems, or combining it with other anti-tumor agents. This approach aims to create third-generation PS based on HPPH, achieving superior biological effects with minimal side effects. This study aims to summarize and present the research and development of third-generation photosensitizers based on HPPH from the last five years, outlining the primary research directions to guide future studies in this domain.

## 2. HPPH-Based Third-Generation Photodynamic Agents

### 2.1. Methods of Modification

#### 2.1.1. Direct Modification of The Basal Structure of HPPH

When designing effective drugs for PDT, the overall lipophilicity of the molecule significantly influences absorption, distribution, metabolism, excretion, and toxicity [25]. The lipid–water partition coefficient (log P) is a vital factor affecting the photodynamic anti-tumor effect [26]. Log P is defined as the logarithm of the ratio between a drug’s equilibrium concentration in octanol and its equilibrium concentration in water. The amphiphilicity of photosensitive materials, characterized by the presence and positioning of hydrophilic and hydrophobic moieties within the molecule, independently interacts with solvents and significantly influences their log P, crucial in tumor uptake. This amphiphilicity also affects the aggregation properties of highly conjugated tetrapyrrole systems, consequently altering the photophysical properties of molecules and their intracellular localization [17]. Enhancements in the lipophilicity of HPPH and its uptake and retention in the tumor microenvironment primarily focus on alterations to its inherent pyrrole ring structure or peripheral modifying groups.

5-Aminolevulinic acid (5-ALA) serves as a prodrug in photodynamic therapy (PDT), enabling the biosynthesis of the photosensitizer protoporphyrin IX (PpIX) within tumors and other rapidly proliferating cellular stages. Additionally, 5-ALA functions as a compound amenable to photosensitizer modification, allowing for modulation of its hydrophilic and hydrophobic properties. Derivatives of 5-ALA, formed through its conjugation with other compounds, possess the potential to generate PpIX upon cellular uptake subsequent to hydrolysis facilitated by cytoplasmic esterases and/or proteases [27]. Gao et al. [20] combined pyropheophorbide a with 5-ALA to produce a series of HPPH derivatives as shown in Figure 2a, to regulate the hydrophilicity and lipophilicity of the photosensitizer, enhancing the compound’s entry into tumor cells. Among these, compounds **5**–**13** represent alkyl ether derivatives with varying lengths of carbon chains; in vitro experiments demonstrated that ether compound’s C chain extension increases its lipid solubility, while the presence of hydroxyl and ether linkages in glycol ether affects the compound’s solubility. The synthesized compounds exhibit strong absorption at 660–670 nm with a high molar absorptivity. The intense absorption in the near-infrared spectrum benefits deep tissue PDT efficacy. In vitro experiments for ROS generation show higher ROS quantum yields for compounds compared to pyropheophorbide A. Incorporating long linear alkyl ether chains in pyropheophoebide-a-ALA conjugation, Compounds **8** and **11** significantly produce singlet oxygen upon laser irradiation at 650 nm, likely due to alterations in the peripheral structure of the tetrapyrrole ring. The tetrapyrrole ring influences the photosensitizer’s aggregation, which reduces ROS quantum yields [28]. Comparing with pristine HPPH as a control, compounds **8**–**11** exhibited notably higher intracellular uptake than other derivatives, and the compounds primarily localized within the cytoplasmic lysosomes, mitochondria, and endoplasmic reticulum in cell experiments using human non-small cell lung cancer cell line A549 cells. Toxicity experiments demonstrated the effective inhibition of tumor cell proliferation by those compounds via the mitochondrial-dependent caspase pathway.

The in vivo PDT efficacy analysis using subcutaneous A549 tumor-bearing BALB/c nude mice models showed significant tumor regression after treatment with compounds **8** and **11**. Even when the treatment dose was halved, efficacy remained. In vivo toxicity experiments with animal subjects receiving compounds **8** and **11** injections higher than therapeutic doses and simulated sunlight exposure did not exhibit significant skin phototoxicity, except for observable skin thickening and edema reactions in rats when doses were increased tenfold. Compounds **8** and **11** demonstrated clinical application potential as photosensitive materials, showing efficient PDT effects without notable skin phototoxicity.

Besides overall lipophilicity, both the chemically reduced ring structure of tetrapyrroles and the presence of certain substituents in the periphery significantly affect the tumor uptake, intracellular localization, and long-term tumor treatment of the agents.

Saenz et al. [21] synthesized HPPH methyl ester (HPPH-ME) and HPPH with chemically reduced ring-B isomers along with methyl ester (iso-HPPH-ME) and carboxylic acid (iso-HPPH) at corresponding positions as shown in Figure 2b. In vitro chemical experiments showed that, compared to HPPH-ME and HPPH, iso-HPPH-ME and iso-HPPH exhibited stronger absorption at ~669 nm, beyond the maximum absorption wavelength of λ_max_ = 660 nm for HPPH, demonstrating enhanced tissue penetration. The singlet oxygen quantum yield of iso-HPPH also slightly increased (~55%) compared to HPPH, exhibiting stable photobleaching properties. In vitro cell experiments using reconstructed co-cultures of stromal cells and cancer cells simulated in vivo tumor tissue, highlighting the cell-type-specific uptake of the PDT agents. Fluorescence analysis revealed HPPH’s binding rate was five times higher than HPPH-ME. High-magnification fluorescence microscopy showed HPPH localized within mitochondria and the endoplasmic reticulum, while HPPH-ME’s fluorescence was present in dense vesicles with a lower degree of localization within mitochondria and the endoplasmic reticulum. HPPH’s retention varied significantly among different cell lines; the head and neck cancer cell line HNT-1 accumulated HPPH at rates three to five times higher than fibroblast cells after a 24-h incubation, whereas the HN-85-1 cell line lost approximately 90% of HPPH within 24 h, similar to the rate observed in stromal cells. In the lung squamous cell carcinoma cell line TEC-1-2, both HPPH and iso-HPPH were absorbed several times faster than their corresponding methyl ester derivatives but also lost from cells at a higher rate. Although the uptake rate of methyl ester derivatives was lower, after continuous incubation for 24 h, the accumulation of HPPH-ME reached a level roughly equal to that of HPPH and iso-HPPH. The stronger retention of methyl ester derivatives might indicate a stronger interaction between the methyl ester group and respective membrane components at subcellular deposition sites. Comparing with iso-HPPH-ME, which contains methyl ester in the B and D ring-reduced tetrapyrrole structure, with HPPH-ME, the cell uptake of iso-HPPH-ME was approximately four times lower, indicating that the porphyrin structure with a chemically reduced B-ring in a neutral charge is less conducive to transmembrane movement [29]. Different reduced ring structures may also have some impact on the efficacy of PDT both in vitro and in vivo. Although similar photodynamic effects were observed in in vitro Colon26 cell experiments, under high light intensity in BALB/c mouse subcutaneous tumor models, HPPH showed slightly better efficacy than iso-HPPH, potentially due to the small group size of only three mice per group, without displaying statistical differences. Both HPPH and iso-HPPH caused vascular damage during light treatment, resulting in an overall decrease in blood flow (~50%), which persisted until the end of the light treatment. The comparison between HPPH and iso-HPPH PDT revealed slight differences in hemodynamics, with iso-HPPH initially causing a rapid increase in blood flow, followed by a gradual decrease. This might be attributed to lower tissue damage induced by iso-HPPH, corresponding to the lower skin phototoxicity displayed by iso-HPPH in PDT side-effect assessments.

Saenz et al. discovered that introducing an alkaline group at position 17^2^ of HPPH enhances the uptake of HPPH by human basal cell carcinoma BCC1 [22]. They synthesized HPPH analogs with varying carboxylic acid functionalities (PS2.3.4), alkyl amines with different lengths of carbon units (PS 5.6), N, N-dimethyl derivatives (PS7), and quaternary ammonium salts (PS8) at position 17^2^ of HPPH as shown in Figure 2c. ABCG2 is an ATP-dependent transporter that effectively transports HPPH. Since the carboxylic acid residue at position 17^2^ converts the pheophorbide to a substrate for the ABCG2-ATP-dependent transporter protein, the experiment used cancer cell lines known to contain high levels of this protein, namely RIF (mouse fibrosarcoma) and human basal cell carcinoma BCC1, to demonstrate the improved cellular uptake of the modified HPPH. Only two compounds, those with amine groups linked to ethylamine (PS 5) or dimethylamine (PS 7), showed absorption rates higher than pristine HPPH. The absorption rate of PS 8, containing a quaternary ammonium salt, was close to that of HPPH, while the absorption rate of PS 6, also containing alkyl amine, was several times lower than HPPH. Compounds with increasing numbers of carboxyl groups at position 17^2^ (PS 2, 3, and 4) showed significantly lower absorption rates than HPPH, which contains only one carboxyl group. After co-incubation of RIF cells with the photosensitizers at 37 °C for 30 min followed by transfer to media without the sensitizer, fluorescence localization showed that most cationic PSs (PS 5, 6, 7, 8, etc.) initially adhered more strongly to the cell surface than pristine HPPH, demonstrating better tumor uptake. In contrast, anionic PSs (PS 2, 3, 4) mainly bound to the cell surface, suggesting that the type, quantity, and structural arrangement of charged groups determine cellular uptake, further influencing the efficacy of PDT in tumors with high levels of the ABCG2 protein [30]. However, in in vivo experiments, even low doses of PS 5 and 8 induced acute injuries such as swelling at the injection site, and higher doses showed accumulation in the liver and kidneys with systemic toxicity. Therefore, increasing the number of aggregated acidic residues enhanced the overall hydrophilicity of HPPH, significantly reducing cellular uptake and retention by tumor cells. Conversely, cationic groups increased cellular binding rates, but this enhanced uptake was accompanied by the loss of PS specificity for tumor cells and severe systemic toxicity, further limiting their clinical application.

#### 2.1.2. Loading HPPH into a Functional Carrier

The encapsulation of HPPH within a functional carrier represents a pivotal strategy in enhancing its therapeutic efficacy. The incorporation of HPPH into specialized carriers offers a multifaceted approach to augment its bioactivity.

**Figure 3:**
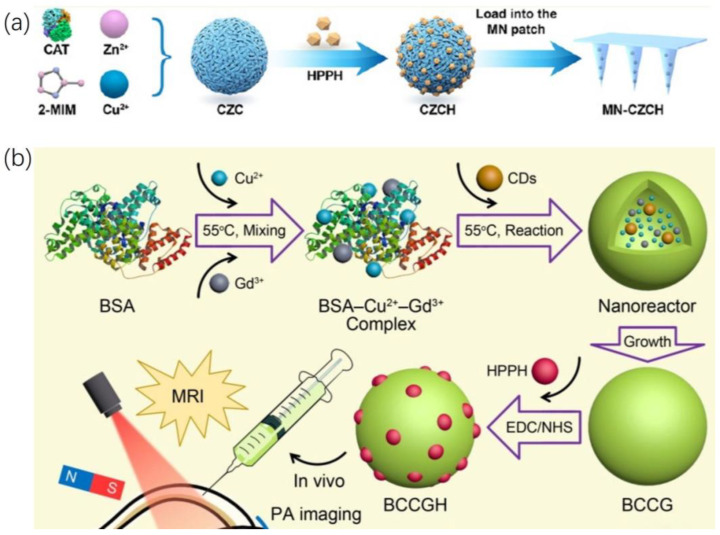
Loading HPPH into a Functional Carrier. (**a**) Cu^2+^-doped zinc imidazolate framework (ZIF) to encapsulate catalase (CAT) and further loaded with HPPH to form a nanomedicine platform CZCH; CZCH was loaded into the MN patch. Reproduced with permission from ref. [31]. Copyright 2022, America Chemistry Society. (**b**) Couple bovine serum albumin (BSA), carbon dots (CDs), and metal ions (Cu^2+^ and Gd^3+^) with the photosensitizer HPPH to synthesize multifunctional nanoparticles (BCCGH). Reproduced with permission from ref. [35]. Copyright 2018, America Chemistry Society.

Li et al. [31] synthesized a microneedle patch suitable for repetitive PDT. The main constituent of the microneedle (MN) patch is hyaluronic acid (HA), known for its excellent tissue compatibility and biodegradability. Comprising a series of micrometer-sized needles, it can painlessly penetrate the epidermis and effectively extend the retention time of drugs within the lesion tissues [32]. In their study, they used a Cu^2+^-doped zinc imidazolate framework (ZIF), known as zinc imidazolate framework with copper (CZ), to encapsulate catalase (CAT) (CZC) and further loaded it with HPPH to form a nanomedicine platform CZCH. This CZCH was then encapsulated into the aforementioned hyaluronic acid microneedles, forming MN-CZCH as shown in Figure 3a. The nanoplatform enhances CAT’s catalytic stability and ensures the slow release of HPPH. CAT catalyzes the conversion of hydrogen peroxide (H_2_O_2_) in the tumor microenvironment (TME) into oxygen (O_2_), providing sufficient substrate for HPPH-mediated PDT, boosting intratumoral reactive oxygen species (ROS) levels under light conditions. Simultaneously, Cu^2+^ mediates glutathione (GSH) depletion to eliminate overexpressed GSH, a strong antioxidant in the TME, further disrupting the abnormal oxidative-reductive state and significantly weakening the PDT resistance of tumor cells [33]. Moreover, repeated treatment guided by HPPH’s intrinsic fluorescence imaging further enhances the anti-tumor effect. Additionally, using this nanoplatform to deliver catalase (CAT) to the local tumor effectively avoids proteinase-induced degradation, instability, and immunogenicity that could arise from exogenous protein entry into the body [34]. In vitro chemical experiments introduced MN-CZCH into phosphate-buffered saline (PBS), measuring changes in absorbance at 660 nm in the supernatant over specific time intervals to detect HPPH content, representing the microneedle’s CZCH release capability. The results indicated sustained release of MN-CZCH in PBS, with an accumulated release rate of 92.3% at 4 h. Cell experiments showed that under both normoxic (21% O_2_) and hypoxic (1% O_2_) conditions, CZCH exhibited higher phototoxicity to A375 cells than HPPH. Correspondingly, the reduction in H_2_O_2_ levels in A375 cells demonstrated that CAT catalyzes the in situ generation of O2. However, the PDT efficacy of HPPH decreased significantly under hypoxic conditions. The CZCH group showed 1.7 times higher intracellular ROS generation than the HPPH group. After co-incubation for 12 h, the GSH content in the CZCH group’s A375 cells reduced by 23.78% compared to the Cu^2+^-free group. For in vivo experiments, BALB/c nude mice were used in a subcutaneous melanoma (A375 cells) xenograft model. MN-HPPH was the control group, and the maximum fluorescence intensity was recorded 4 h after administration. Subsequently, HPPH rapidly cleared from the tumor tissue. In contrast, while the FL signal of MN-CZCH was weaker than the control group within 4 h post-administration, over time, the FL signal continued to increase and reached its maximum at 24 h, six times stronger than the maximum fluorescence signal of the MN-HPPH group. Results indicated that the tumor growth inhibition rates for MN-HPPH(+) and MN-CZCH(+) groups were 82.7% and 97.7%, respectively. All mice treated with a single laser irradiation (30 min, 100 mW/cm^2^) exhibited signs of tumor recurrence in the second week, showing significantly inferior efficacy compared to the repeated treatment group, where mice were treated again for 30 min with a laser after 24 h of MN-CZCH treatment. Hence, the CZCH nanoplatform enhances HPPH-mediated tumor ablation through self-supplied O_2_, Cu^2+^-mediated GSH depletion, and repeated irradiation.

Loading HPPH into liposomes or nanomaterials enhances its biocompatibility, favoring drug distribution or excretion. Additionally, these materials can simultaneously encapsulate other effective ingredients or be designed to achieve photo-thermal/photoacoustic/photodynamic multimodal actions under suitable light conditions. Current research and development in carrier materials have significantly boosted the efficacy of HPPH in PDT and PDT combined with radiotherapy, chemotherapy or immunotherapy.

Hua et al. [35] synthesized multifunctional nanoparticles (designated as BCCGH) by coupling bovine serum albumin (BSA), carbon dots (CDs), and metal ions (Cu^2+^ and Gd^3+^) with the photosensitizer HPPH, resulting in ultra-small-sized (~7.9 nm) entities as shown in Figure 3b. BSA, with inherent tissue compatibility, and the ultra-small size, reduce adverse clearance by the reticuloendothelial system, enabling deeper tumor penetration and targeting of cell nuclear structures more sensitive to ROS loss damage. BCCG, with superior PT properties, was synthesized through a 6-h reaction of BSA–metal ions (Cu^2+^ and Gd^3+^) precursor and CDs, exhibiting excellent photothermal conversion efficiency (68.4% at 808 nm). Coupling with HPPH achieved nucleus-targeted PTT-triggered PDT. In vitro and in vivo studies conducted on human lung cancer (A549) cells revealed that BCCGH largely avoided entrapment in lysosomes or endosomes and primarily accumulated in the endoplasmic reticulum surrounding the cell nucleus. Upon low-power near-infrared laser irradiation (808 nm, 0.3 W cm^–2^), the tumor site’s temperature increased by 9.2 °C (from 33.9 °C to 43.1 °C), exhibiting enhanced cell uptake and nucleus-targeting capabilities. The high photothermal conversion efficiency and tumor enrichment of BCCGH, coupled with low drug dosages and laser power, sufficiently and safely increased the temperature at the tumor site, inducing ROS production within the cell nucleus upon subsequent 671 nm laser irradiation, further enhancing PDT efficacy.

Control groups treated solely with PTT or PDT, despite the excellent tumor-targeting capabilities of BCCGH, exhibited unsatisfactory tumor ablation efficiency after a single session of PTT or PDT, suggesting insufficient temperature elevation (with low-power lasers) or ROS generation during PDT to eradicate the tumor. In vivo imaging experiments on tumor-bearing nude mice showed BCCGH’s high longitudinal relaxivity (11.84 mM^–1^ s^–1^, 7 T) and exceptional colloidal stability (negligible Gd^3+^ release), enabling multimodal imaging-guided synergistic PTT/PDT.

Ex vivo fluorescence imaging of major organs (heart, liver, spleen, lungs, and kidneys) and tumor tissue in tumor-bearing mice revealed that one day post-injection, BCCGH mainly accumulated in the liver, tumor, and kidneys, potentially due to its ultrasmall hydrodynamic diameter (6.5 ± 1.1 nm) [36] and high levels of lipoprotein receptor expression in these organs [29]. The Cu contents in the collected samples were measured by ICP-OES, indicating that BCCGH was effectively excreted from the organism primarily via urine or feces after seven days.

### 2.2. Combinatorial Treatment of HPPH and Other Materials

By modifying the inherent structure of HPPH to incorporate other functional groups or loading drugs with different anti-tumor mechanisms alongside HPPH into carrier materials, various modes of targeted multi-mechanism anti-tumor effects can be achieved, ultimately resulting in synergistic effects greater than the sum of the individual parts.

**Figure 4:**
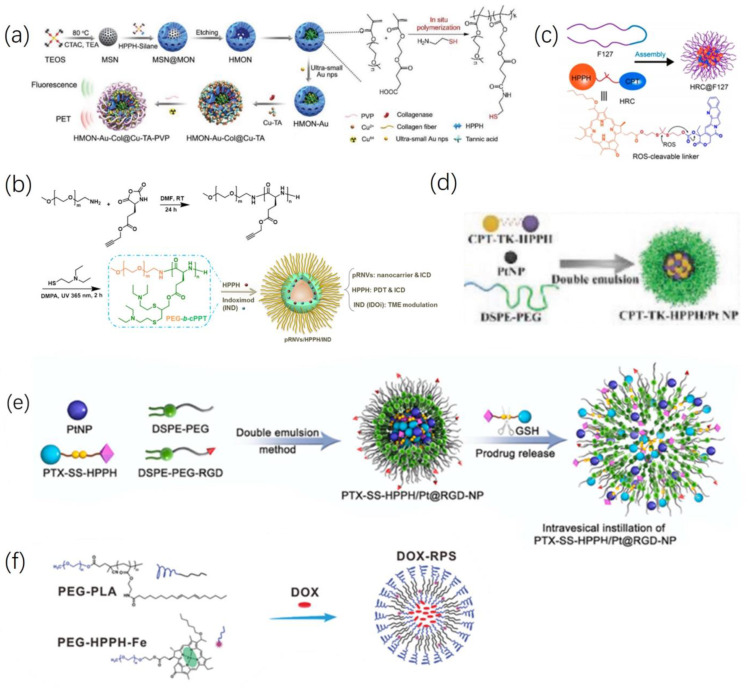
Combinatorial Treatment of HPPH and Other Materials. (**a**) “In situ framework growth” approach to construct HMONs hybridized with the HPPH, then polymer-containing thiol groups can be trapped in the hollow cavity to immobilize the Au NPs via the chelation effect. TA was deposited on the surface of the HMONs to initiate the coordination with the metal ions Cu^2+^. Reproduced with permission from ref. [40]. Copyright 2020, Wiley. (**b**) Construction of pH-responsive nanovesicles (pRNVs/HPPH/IND) via co-assembly of HPPH, IND, and pH-responsive polypeptide. Reproduced with permission from ref. [41]. Copyright 2022, America Chemistry Society. (**c**) Scheme of prodrug HRC encapsulated by F127 (HRC@F127 nanoparticles), which is activated by reactive oxygen species (ROS). Reproduced with permission from ref. [47]. Copyright 2020, America Chemistry Society. (**d**) ROS-responsive prodrug (CPT-TK-HPPH) that consisted of thioketal bond linked CPT and photosensitizer HPPH, loaded to PtNP (CPT-TK-HPPH/Pt NP). Reproduced with permission from ref. [46]. Copyright 2020, Wiley-VCH GmbH. (**e**) A novel RGD peptide modified PtNP coloaded GSH-responsive prodrug nanoparticles (PTX–SS–HPPH/ Pt@RGD-NP). Reproduced with permission from ref. [48]. Copyright 2023, Elsevier. (**f**) A doxorubicin hydrochloride (DOX)-loaded ROS-responsive polymersome (DOX-RPS) is prepared via the self-assembly of amphiphilic poly (ethylene glycol)-poly (linoleic acid) (PEGPLA) and poly (ethylene glycol)-(2-(1-hexyloxyethyl)-2-devinyl pyropheophorbide-α)-iron chelate (PEG-HPPH-Fe). Reproduced with permission from ref. [50]. Copyright 2021, Wiley-VCH GmbH.

Although tumor tissues contain a higher level of ROS compared to normal tissues, the unique redox balance systems in tumors prevent excessive ROS from damaging tumor cells. However, within tumor cells, ROS primarily exists in the form of low-toxicity H_2_O_2_. Converting these species into highly toxic ^•^OH radicals could disrupt the redox equilibrium within the tumor microenvironment, enabling a cytotoxic effect against tumor cells [37]. Chemodynamic therapy (CDT) involves the utilization of Fenton or Fenton-like reactions to convert hydrogen peroxide (H_2_O_2_) into hydroxyl radicals (^•^OH), one of the most potent reactive oxygen species (ROS) [38]. Unlike PDT, CDT is independent of localized oxygen concentrations. It harnesses tumor-specific endogenous chemical energy to generate ^•^OH, mitigating oxidative damage to normal tissues. However, unfortunately, despite the relatively elevated levels of H_2_O_2_ in the tumor microenvironment (TME) compared to normal tissues, the endogenous quantity of H_2_O_2_ remains insufficient to achieve effective CDT [39]. In a study conducted by Li et al. [40], they constructed hollow mesoporous organosilica nanoparticles (HMONs) interwoven with the HPPH through an “in situ framework growth” approach. These HMONs, serving as nanoreactors for “in situ polymerization,” facilitated the synthesis of thiol-functionalized polymers, thereby anchoring gold nanoparticles (Au NPs). The ultra-small (<3.6 nm) Au NPs acted as glucose oxidase-like nanozymes efficiently catalyzing the conversion of glucose into H_2_O_2_ and gluconic acid. Subsequently, Cu-TA complexes were further deposited onto the HMON surface (HMON-Au@Cu-TA) to initiate a Fenton-like reaction, converting the provided H2O2 into the highly toxic ROS, ^•^OH. To enhance colloidal stability during systemic circulation, polyvinylpyrrolidone (PVP) was coated onto HMON-Au@Cu-TA, forming HMON-Au@Cu-TA-PVP. Finally, collagenase targeting extracellular matrix type I collagen fibers (Col) was loaded into HMON-Au@Cu-TA-PVP, enhancing nanoparticle accumulation and penetration in tumor tissues, independent of tissue oxygen levels. This engineered biocatalytic nanoreactor, HMON-Au-Col@Cu-TA-PVP, as shown in Figure 4a, amalgamates the synergistic effects of PDT/CDT. Experimental evidence, both in vitro and in vivo using BxPC-3 cells (human pancreas cancer cells), demonstrates that this material induces apoptosis in tumor cells and partially retards tumor growth under non-illuminated conditions. Under light exposure, HMON-Au@Cu-TA-PVP achieves the synergistic PDT and CDT effects, bolstering the ROS-mediated anti-pancreatic ductal adenocarcinoma effects, resulting in rapid tumor regression and ~80% long-term tumor-free survival in mice.

Yang et al. [41] discovered that pH-responsive nanovesicles (pRNVs), self-assembled from polyethylene glycol-b-cationic polypeptide (PEG-b-cPPT) block copolymers, function not only as nano-carriers but also induce immunogenic cell death (ICD) via calreticulin (CRT) exposure preceding apoptosis. Co-encapsulation of the photosensitizer HPPH and indoleamine 2,3-dioxygenase inhibitor, indoximod (IND), within single low-dose pRNVs/HPPH/IND as shown in Figure 4b, exhibited remarkable PDT and immunologically enhanced anti-tumor efficacy with good biocompatibility upon laser irradiation in the B16F10 melanoma model. The research suggests that in acidic environments, the protonation of tertiary amines in double-loaded pRNVs (pRNV/HPPH/IND) generates a positive charge within the endosomes, prompting endosomal escape of pRNVs, subsequently releasing HPPH and IND into the cytoplasm. Cell experiments observed similar intracellular HPPH fluorescence intensities between free HPPH and various HPPH-loaded nanoformulations in B16F10 cells after 4 h of co-incubation. Prolonging incubation to 24 h showed similar intracellular HPPH fluorescence intensities across all groups, indicating uptake and internalization predominantly occur within the initial 4 h. Fluorescence co-localization analysis revealed partial uptake of pRNVs by B16F10 cells at 0.5 h, with limited co-localization (weak red fluorescence of HPPH) with lysosomes; more co-localization (appearing yellow) at 1 h indicated pRNVs being trapped within the endosomal/lysosomal compartment, followed by their escape by 2 h, displaying separate red and green signals, suggesting possible re-release of loaded drugs from lysosomes into the cytoplasm. IND, through phosphorylating P-S6K, restores the mTOR pathway, regulating the tumor microenvironment (TME), ultimately stimulating CD8+ T cell development for tumor immunotherapy. However, individual action of IND alone hardly caused cell death (11.6%). Under 671 nm laser irradiation (100 mW/cm^2^, 1 min), free HPPH (39.4%) and HPPH/IND (36.4%) predominantly induced early apoptosis without the pRNVs carrier. Both early and late apoptosis were observed in pRNVs/HPPH (20.6%, 33.6%) and pRNVs/HPPH/IND (10.1%, 45.6%)-treated B16F10 cells, where the addition of IND caused more late-stage apoptosis. Supplementary experiments indicated weak induction of CRT expression by pRNVs in MC38 and 4T1 cells and no significant ICD, suggesting cell-dependent effects of pRNVs-induced ICD. The data hinted that B16F10 cells might be more sensitive to ICD exerted by pH-responsive tertiary amines or thiol ether groups through CRT exposure. In vivo experiments on C57BL/6 mice with subcutaneous B16F10 tumors revealed that after laser irradiation of pNRV/HPPH/IND, blood cytokine levels (such as IL-6, TNF-α) were higher than in the free HPPH group at 24 and 48 h. The pRNVs/HPPH/IND-treated group displayed the most infiltration of CD8+ T cells in primary and distant tumor tissues compared to other groups. Additionally, the ratio of CD8+ T cells to CD4+ T cells in tumors was higher in the pRNVs/HPPH/IND group than in the pRNVs/HPPH group, suggesting IND addition could reverse the tumor microenvironment, activating CD8+ T cells. pRNVs/HPPH notably suppressed primary and distant tumor growth within 17 days post-tumor inoculation, yet due to immune-suppressive factors and the presence of Treg cells, this later weakened the activation of CD8+ T cells, resulting in tumor regrowth in pRNVs/HPPH-treated mice between days 13 to 17 post-inoculation of tumors. Individual use of free IND similar to the PBS control hardly exhibited any inhibitory effect on tumor growth. However, combining pRNVs/HPPH with low-dose IND as pRNVs/HPPH/IND significantly reduced tumor growth by activating CD8+ T cells and alleviating immune-suppressive effects. H&E staining observed prominent tumor cell death in pRNVs/HPPH/IND-treated mice without apparent organ damage, indicating the nano-platform’s good safety profile.

Currently, the most prevalent systemic anti-tumor approach involves chemotherapy, with the combination of chemotherapy and PDT being extensively researched as a combined therapeutic model. Epirubicin (EPI), a broad-spectrum anticancer agent, is commonly employed in the chemotherapeutic treatment of osteosarcoma [42]. Graphene oxide (GO), recognized as the thinnest nanomaterial, due to its excellent biocompatibility, finds application in drug delivery [43]. Cell-penetrating peptides (CPPs), low-molecular-weight peptides possessing membrane translocation abilities, serve as carriers facilitating the cellular entry of various large molecules [44]. Zhang et al. [45] combined these aforementioned materials, using polyethylene glycol (PEG) linkage to produce cell-penetrating peptide (CPP)-modified graphene oxide (pGO). They loaded HPPH and EPI onto this material, forming HPPH/EPI/CPP-pGO, significantly enhancing drug delivery to tumor tissues. As compared to free HPPH, CPP and pGO-loaded HPPH exhibited markedly increased cellular uptake and ^1^O_2_ generation, with lower cytotoxicity under dark conditions. Similarly, EPI/CPP-pGO treatment inhibited cell viability and colony formation, inducing apoptosis. Moreover, HPPH/EPI/CPP–pGO achieved a pronounced synergistic effect by combining PDT and chemotherapy, resulting in the most conspicuous suppression of tumor cell proliferation. In vivo experiments, utilizing a xenograft model with subcutaneously implanted MG-63 cells, corroborated these findings, demonstrating the most effective tumor growth inhibition in the HPPH/EPI/CPP–pGO treatment group.

Hao et al. [46] synthesized a material that enables ROS-mediated release of chemotherapeutic drugs. This material comprises ROS-reactive prodrugs formed by linking camptothecin (CPT) and HPPH via thioketal (TK) bonds. Loaded into platinum nanozymes (Pt NP) with hydrogen peroxide (H_2_O_2_) enzymatic activity, and further enveloped by amphiphilic polymers distearylphosphatidylethanolamine–polyethylene glycol (DSPE-PEG) as shown in Figure 4d. Upon 660 nm laser irradiation, Pt NP decomposes H_2_O_2_ within the tumor microenvironment, generating oxygen to fuel HPPH-mediated PDT, leading to TK bond cleavage, thereby releasing CPT. This photodynamic-induced combined chemotherapy effectively inhibits tumor proliferation and growth both in vitro and in vivo. In a similar vein, Jiang et al. [47] studied a nanomaterial with the same active components, CPT-TK-HPPH (referred to as HRC in their study). They employed the biocompatible polymer Pluronic F127 to encapsulate the HRC as shown in Figure 4c, demonstrating aggregation-induced fluorescence quenching (ACQ) due to strong π−π stacking within the nanoparticles. Upon internalization into cells, HRC NPs are activated by endogenous ROS in the tumor microenvironment, releasing free CPT and HPPH molecules, facilitating tumor-specific fluorescence imaging of HPPH, PDT, and chemotherapy collaboration. Another material, PTX-SS-HPPH /Pt@RGD-NP synthesized by Hao et al. [48], as shown in Figure 4e, targets the antioxidant component glutathione (GSH) within the TME, releasing paclitaxel (PTX) and HPPH via GSH-mediated disulfide bond cleavage. Additionally, the outer structure of this material incorporates an Arg-Gly-Asp (RGD)-modified platinum nanozyme (PtNP), which specifically binds to integrin αvβ3 on tumor cell surfaces, enabling tumor targeting. Moreover, it utilizes neuropilin-1 to enhance tissue penetration via the C-end rule (CendR) [49]. Successfully achieving GSH-responsive drug release in human bladder cancer (T24) cells, this Pt@RGD-NP structure improves drug penetration into tumor cells, prolongs retention at tumor sites, alleviates tumor tissue hypoxia, and enhances the efficacy of PDT in combination with chemotherapy.

Wang et al. [50] further proposed a more complex, amphiphilic poly(ethylene glycol)-poly(linoleic acid) (PEG-PLA) and poly (ethylene glycol)-(2-(1-hexyloxyethyl)-2-devinyl pyropheophorbide-α)-iron chelate (PEG-HPPH-Fe) self-assembly strategy to generate a doxorubicin hydrochloride-loaded ROS-responsive polymersome (DOX-RPS) as shown in Figure 4f. This polymer employed a photodynamic–chemodynamic cascade strategy, enabling ROS-triggered drug release while simultaneously regenerating ROS, circumventing ROS depletion during drug release and offering new prospects for enhancing the combined efficacy of photodynamic and chemical therapies. Upon laser irradiation, photosensitizer HPPH effectively generated primarily ^1^O_2_-mediated ROS, leading to the in-situ oxidation of linoleic acid chains within PLA to form linoleic acid peroxides (LAP). The presence of hydrophilic peroxide groups within LAP altered the permeability and structural stability of the polymer, causing disruption in the RPS-like cell membrane structure and triggering drug release. Subsequently, catalyzed by HPPH-Fe, LAP regenerated ROS through a chemodynamic process. In vitro control experiments highlighted the crucial role of Fe in facilitating the Fenton reaction. Cellular experiments with U87MG cells demonstrated superior anti-tumor efficacy and longer mouse survival periods, while the tumor-specificity and biosafety were superior, as evidenced by PET imaging and weight monitoring.

As the duration or frequency of PDT treatments increases, a further reduction in oxygen levels within the tumor microenvironment (TME) leads to diminished ROS production, resulting in PDT resistance. In such cases, certain hypoxia-activated prodrugs can leverage the severe hypoxic conditions caused by PDT to enhance the efficacy of combined therapy [51]. Triple-negative breast cancer (TNBC) is characterized by the absence of estrogen receptors (ER), progesterone receptors (PR), and human epidermal growth factor receptor (HER2), leading to high failure rates in traditional treatments [52]. Clinical studies involving 139 cases of breast invasive ductal carcinoma (BIDC) indicated that TNBC exhibits overexpression of CD44 compared to non-TNBC cases [53]. CD44 (cluster of differentiation-44) is an adhesion molecule associated with tumor occurrence and metastasis, effectively targeted by chitosan oligosaccharide (CO) [54]. Building upon this understanding, Ding et al. [55] co-encapsulated HPPH and the hypoxia-activated prodrug, TH302, within the hydrophobic bilayer structure of liposomes. They surface-modified the liposomes by linking CO to achieve targeted uptake by CD44+ tumor cells. The CO-HPPH-TH302/Lipo formulation demonstrated PDT efficacy under light conditions while effecting hypoxia-activated chemotherapy. Both in vitro and in vivo experiments showcased significantly superior anti-tumor effects compared to monotherapy groups.

### 2.3. Combined Tumor Imaging and Treatment

During photodynamic reactions, certain photosensitizers can also react with generated singlet oxygen (^1^O_2_), resulting in decreased photosensitizer concentration, absorbance, and fluorescence intensity. This phenomenon is termed photobleaching [56]. Due to the spectral characteristics of HPPH absorption, exposure to visible light after injection or fluorescence imaging prior to treatment may prompt the photosensitizer (PS) to reach an excited state prematurely, before its intended therapeutic activation. This premature activation, causing photobleaching, can to some extent diminish the efficacy of photodynamic therapy. Improvements in photosensitizers allow for non-invasive imaging and reassessment of tumors prior to PDT without compromising the photosensitizer’s properties or PDT efficacy. Additionally, these enhancements enable non-invasive assessment of the systemic distribution of photosensitive materials after entering the body and the evaluation of local and systemic tumor efficacy post-treatment.

#### 2.3.1. Fluorescence Imaging

**Figure 5:**
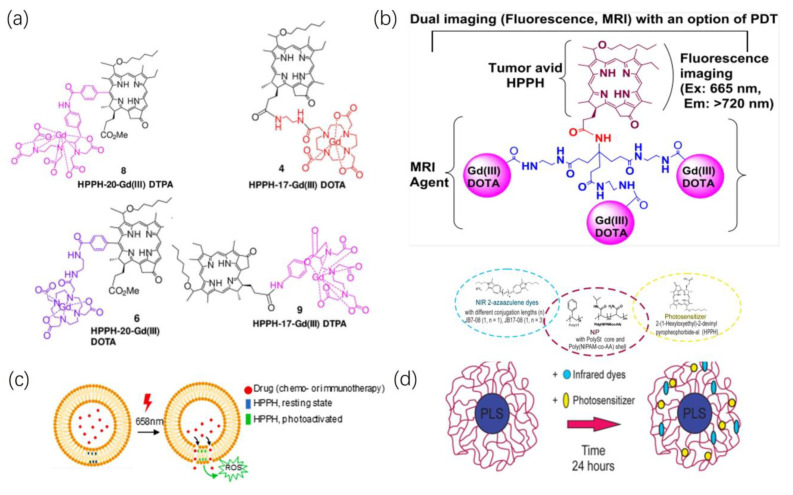
Imaging. (**a**) HPPH was conjugated at various peripheral positions (position 17 or 20) with either Gd(III) DTPA or Gd(III) DOTA. Reproduced with permission from ref. [62]. Copyright 2020, John Wiley and Sons, Inc. (**b**) HPPH conjugates with tri-Gd(III)DTPA, the HPPH-3Gd(III)DTPA. Reproduced with permission from ref. [63]. Copyright 2022, America Chemistry Society. (**c**) Schematic illustrating liposomal release of reactive oxygen species (ROS) on exposure to 660 nm laser light. Reproduced with permission from ref. [24]. Copyright 2023, Elsevier. (**d**) Chemical structures of NIRF dyes (left, circled by blue dashed line), polymers that form polySt-poly(NIPAM-co-AA) NPs (central, circled by purple dashed line) and photosensitizer (right, circled by yellow dashed line), and preparation of nanoformulations. Reproduced with permission from ref. [61]. Copyright 2020, BioMed Central.

Zenga et al. [24] developed light-activated HPPH liposomes containing trace amounts of a near-infrared lipid probe, DiR (Ex/Em 785/830 nm), as shown in Figure 5c. Leveraging the tumor’s enhanced permeability and retention (EPR) effect, these liposomes demonstrated passive selective accumulation within tumors. In in vivo experiments, the concentration peaked at 4 h post intravenous injection, with the inactive HPPH residing within the bilayer structure of the liposomes. Upon light exposure, the activation of HPPH induced ROS production, disrupting the liposomal structure and leading to the release of encapsulated DiR. This facilitated the assessment of extracellular tumor leakage within the tumor microenvironment and accumulation in vital organs post-PDT therapy. This straightforward material design not only offers a feasible non-invasive method for assessing the distribution or excretion of photosensitive materials post-treatment but also paves the way for liposomal co-delivery of drugs in PDT combined with other targeted cancer therapies.

When evaluating the biological distribution of PS through fluorescence imaging, the excitation of PS can result in premature photobleaching and toxicity to healthy tissues. The coupling of PS with separate fluorescence segments allows for excitation outside of PS activation, potentially enabling the delivery of PS to cancer sites under fluorescence imaging guidance for subsequent PDT. Near-infrared fluorescent dyes (NIRFD) utilized to label PS fragments facilitate NIR fluorescence imaging to guide PS delivery for phototherapy and/or fluorescence-guided surgery [57]. However, the coupling of PS with traditional near-infrared dyes may diminish PDT efficacy due to undesirable electronic excitation energy transfer between PS and these dyes, known as Förster resonance energy transfer (FRET), impacting both the pharmacokinetics of PS and diminishing its efficacy [58,59]. Studies involving HPPH-NIRFD conjugates demonstrate that electronic excitation energy transfer between HPPH and near-infrared fluorescent dyes leads to a significantly lower singlet oxygen yield compared to when HPPH is in-dependently excited [59]. Various studies on HPPH and anthocyanin dye conjugates illustrate that the orientation of two emitting groups and their connecting linker length influence FRET efficiency, significantly impacting in vivo PDT outcomes. Recently detected optical windows in the SWIR region (~1000–1700 nm) for in vivo optical imaging show reduced tissue scattering and autofluorescence, allowing higher-resolution imaging of deeper tissues [60]. Consequently, Chepurna et al. [61] synthesized core–shell polymeric nanoparticles (NPs) with a polystyrene (polySt) core and a shell composed of a copolymer of N-isopropylacrylamide and acrylamide (polySt-poly(NIPAM-co-AA)) as a nanoplatform for NIRFD/PS combinations as shown in Figure 5d. These nanoparticles allow for the encapsulation of hydrophobic molecules such as HPPH and NIRFD, preventing aggregation and reducing fluorescence quenching and singlet oxygen generation. These nanoparticles of different sizes (NPs_D1 and NPs_D2) were synthesized, utilizing two different lengths of 2-azaazulene dyes (JB7-08 and JB17-08) as NIRFD. The overlap between JB7-08 and HPPH’s emission spectra results in stronger electronic excitation energy transfer, affecting the fluorescence intensity of HPPH in NF. In contrast to HPPH, the fluorescence intensity of NIRFD in NF relies on the size of the nanoparticles, with larger NPs (NPs_D2) showing higher fluorescence intensity due to a thicker shell enabling more effective NIRFD loading. PDT and imaging experiments demonstrated superior performance using NPs_D2-based NF com-pared to NPs_D1. The presence of HPPH in NFs results in lower singlet oxygen generation than NFs loaded solely with HPPH, confirmed by fluorescence imaging and cellular experiments. Additionally, cell uptake studies using HeLa cells show a significant difference between HPPH/JB7-08 and HPPH/JB17-08 NFs, with HPPH/JB17-08 NF exhibiting increased necrosis after laser exposure. In vivo fluorescence imaging indicates prolonged fluorescence signals in tumor areas following NF injection compared to free HPPH, indicating the potential for targeted therapy. In summary, employing dyes with absorption spectra close to HPPH may induce adverse FRET, causing premature photobleaching and reducing PDT efficacy. Using longer-wavelength-absorbing NIRFD favors the efficiency of PS/NIRFD NFs in PDT, enabling NIR-II fluorescence, enhancing deep tissue imaging, and reducing tissue scattering and autofluorescence, thereby improving fluorescence imaging contrast, sensitivity, and penetration depth.

#### 2.3.2. Magnetic Resonance Imaging

In addition to simple fluorescence imaging, a combination of various imaging modalities can be achieved through material enhancements. Currently, the most widely used clinical imaging method for soft tissues with relatively high sensitivity is magnetic resonance imaging (MRI). MRI does not have practical depth limitations for in vivo penetration, presenting a significant advantage over optical fluorescence and ultrasound imaging. However, MRI suffers from lower imaging sensitivity and higher costs [6]. Within the near-infrared range of the electromagnetic spectrum, optical fluorescence imaging (OFI) can partially compensate for these shortcomings of MRI. OFI offers rapid data acquisition, high sensitivity, and cost-effectiveness. Therefore, using the information provided by optical fluorescence imaging to complement MRI’s high-resolution anatomical imaging can further expand the clinical applications of both modalities and provide a more comprehensive overview of disease states [7,8].

Zhang et al. [62] synthesized four conjugates as shown in Figure 5a, by linking Gd(III) DOTA (Dotarem) and DTPA (Magnevis) to the 17th and 20th positions of the HPPH fragment, respectively. They further compared their combined MR and fluorescence imaging capabilities, along with the differences in PDT efficacy. In BALB/c mice implanted with Colon-26, MRI exhibited higher T1 relaxivity for the DOTA conjugate compared to the DTPA conjugate. Under similar tissue depositions, this suggests a more substantial signal enhancement for T1-weighted imaging. Moreover, the study demonstrated the higher deposition of HPPH-17-Gd(III) DOTA within the tumor, confirming the significant impact of DOTA as a chelating agent for Gd on the ability of HPPH conjugates as MRI contrast agents. In vivo PDT efficacy experiments involved intravenous injection of [HPPH-17-Gd(III) DOTA]P at MRI imaging doses, followed by light exposure (wavelength 665 nm, 40 J/cm^2^, 40 mW/cm^2^) after 48 h. Within 60 days post-treatment, 10 tumor-bearing BALB/c mice displayed no significant adverse effects, and 70% of the mice exhibited visible tumor regression, which indicated that DOTA-conjugated HPPH represents a multifunctional drug with substantial potential for broader clinical applications.

Cheruku et al. [63] investigated the impact of conjugating different quantities of Gd(III) DOTA at position 17^2^ of HPPH as shown in Figure 5b, on tumor uptake, pharmacokinetics, fluorescence, and MR imaging capabilities. With an increase in the Gd-DOTA conjugates, the compound’s T1 relaxivity showed an almost linear increase, surpassing the reported relaxivity of clinically available contrast agent Gd-DOTA. Furthermore, as the Gd-DOTA groups conjugated with HPPH increased, the T2/T1 relaxivity ratio (r_2_/r_1_) decreased. This decrease could potentially be attributed to the increased hydrophilicity of the compound due to the augmented DOTA groups, thereby reducing the potential aggregation of the porphyrin ring. Further animal experiments also evidenced the biosafety and PDT efficacy of such conjugates, suggesting the promising application prospect of HPPH-based tumor imaging in the future [64].

## 3. Conclusions

The improvement in HPPH’s structure is mainly focused on the peripheral structure of its tetrapyrrole framework, resulting in enhanced biological effects, primarily in the photodynamic sensitizer’s lipophilicity. This elevation in lipophilicity enhances cellular uptake and the quantity of drug metabolism and/or excretion. Observing various material synthesis processes reported in studies, intermediate products during the synthesis of materials with complex structures seem to progressively improve the optical physical properties compared to HPPH. However, due to HPPH’s inherent hydrophobic nature, simple structural alterations often do not yield biological effects in animal models as outstanding as those demonstrated in physicochemical tests or in vitro cell experiments.

Regarding drug delivery systems, current research is focusing on the development of organic molecular frameworks and various novel nanomaterials. Homogeneously dispersing inorganic, organic, or hybrid nanoscale components within a polymeric matrix results in polymer nanocomposites that exhibit distinct physical and/or chemical phases. Polymer/metal organic framework (MOF) nanocomposites are among the extensively used composite materials for drug delivery and imaging applications. The long-term stability of carriers determines their storage duration, indirectly reflecting their clinical potential, a fact that merits inclusion in carrier platform research. Ballav M. Borah and colleagues [65] have researched a novel freeze-dried, biodegradable, and non-toxic amine-functionalized polyacrylamide (AFPAA) nanoparticle formula. They have demonstrated that this freeze-dried formulation can store for a significant duration without significant loss of HPPH’s effective payload. The increasing development of carrier platforms capable of efficiently loading various drugs while maintaining long-term stability will lay the groundwork for enhancing PDT efficacy.

The multifunctional nature of third-generation PS primarily manifests in their imaging localization capabilities and combined therapeutic potential. While HPPH possesses absorption and emission spectra suitable for imaging, utilizing HPPH itself for imaging inevitably triggers photobleaching, diminishing the efficacy of PDT. Currently, the approach mainly involves combining other fluorescent groups or contrast agents to circumvent premature photobleaching of the photosensitizer, thereby mitigating its impact on therapeutic outcomes. Current research on combined therapies focuses primarily on PDT combined with chemotherapy, leveraging PDT’s targeting ability to reduce systemic toxicity associated with chemotherapy. Achieving localized therapy also provides conditions for more precise cellular localization of photosensitizers, thereby realizing superior cumulative biological effects through optimized multi-therapeutic interventions.

## 4. Future Perspectives

As the structure of photosensitizers becomes increasingly intricate and encompasses more unique components with effective properties, it is becoming more pertinent to shift focus from theoretical research based on single components to evaluating the overall effects of materials within biological systems, particularly their potential manifestations within the human body. This encompasses both imaging and therapeutic effects. While enhancing and expanding material properties, interactions or mutual influences between various components are inevitable. For instance, stronger tumor accumulation may enhance anti-tumor efficacy, yet it might reduce imaging sensitivity due to relatively higher accumulation in other areas or introduce noticeable systemic toxicity. Hence, while researching or synthesizing more potent multifunctional photosensitizers, maintaining a balance among their various properties is crucial to achieve the desired overall therapeutic outcome. With continuous advancements in imaging technologies and the emergence of new immunotherapies and targeted treatments, it is crucial to enhance the focus on these pertinent fields and regularly update strategies for the development and research of multi-functional photosensitizers.

Finally, although developing efficient photosensitizers remains the primary approach to enhancing PDT efficacy, future photodynamic research should not solely focus on material development and improvement. Auxiliary therapeutic methods, such as enhancing resistant tumors’ uptake through physical electroporation [66], more precise and uniformly distributed therapeutic light technologies [67], tailoring therapies for different cancer types [68], personalized treatment plans for patients, and rigorous, comprehensive studies to identify measurable indicators during photodynamic therapy, all demand more rigorous and profound investigation.

## Data Availability

All the data are publicly available online.

## References

[1] Dabrowski J.M., Arnaut L.G. (2015). Photodynamic therapy (PDT) of cancer: From local to systemic treatment. Photochem. Photobiol. Sci..

[2] Lee C.-N., Hsu R., Chen H., Wong T.-W. (2020). Daylight Photodynamic Therapy: An Update. Molecules.

[3] Ackroyd R., Kelty C., Brown N., Reed M. (2001). The History of Photodetection and Photodynamic Therapy. Photochem. Photobiol..

[4] Mitton D., Ackroyd R. (2008). A brief overview of photodynamic therapy in Europe. Photodiagnosis Photodyn. Ther..

[5] Sarbadhikary P., George B.P., Abrahamse H. (2021). Recent Advances in Photosensitizers as Multifunctional Theranostic Agents for Imaging-Guided Photodynamic Therapy of Cancer. Theranostics.

[6] Felsher D.W. (2003). Photodynamic therapy for cancer. Nat. Rev. Cancer.

[7] Chen D., Xu Q., Wang W., Shao J., Huang W., Dong X. (2021). Type I Photosensitizers Revitalizing Photodynamic Oncotherapy. Small.

[8] Agostinis P., Berg K., Cengel K.A., Foster T.H., Girotti A.W., Gollnick S.O., Hahn S.M., Hamblin M.R., Juzeniene A., Kessel D. (2011). Photodynamic therapy of cancer: An update. CA Cancer J. Clin..

[9] Correia J.H., Rodrigues J.A., Pimenta S., Dong T., Yang Z. (2021). Photodynamic Therapy Review: Principles, Photosensitizers, Applications, and Future Directions. Pharmaceutics.

[10] Calixto G., Bernegossi J., de Freitas L., Fontana C., Chorilli M. (2016). Nanotechnology-Based Drug Delivery Systems for Photodynamic Therapy of Cancer: A Review. Molecules.

[11] Li G., Wang Q., Liu J., Wu M., Ji H., Qin Y., Zhou X., Wu L. (2021). Innovative strategies for enhanced tumor photodynamic therapy. J. Mater. Chem. B.

[12] Baskaran R., Lee J., Yang S.-G. (2018). Clinical development of photodynamic agents and therapeutic applications. Biomater. Res..

[13] Allison R.R., Bagnato V.S., Cuenca R., Downie G.H., Sibata C.H. (2006). The future of photodynamic therapy in oncology. Future Med..

[14] Chilakamarthi U., Giribabu L. (2017). Photodynamic Therapy: Past, Present and Future. Chem. Rec..

[15] Mfouo-Tynga I.S., Dias L.D., Inada N.M., Kurachi C. (2021). Features of third generation photosensitizers used in anticancer photodynamic therapy: Review. Photodiagnosis Photodyn. Ther..

[16] Hashimoto Y., Naito K., Tsutsumi J. (1962). Photosensitization of animals by the viscera of abalones, haliotis spp. Jpn. J. Med. Sci. Biol..

[17] Ethirajan M., Chen Y., Joshi P., Pandey R.K. (2011). The role of porphyrin chemistry in tumor imaging and photodynamic therapy. Chem. Soc. Rev..

[18] Bellnier D.A., Greco W.R., Loewen G.M., Nava H., Oseroff A.R., Pandey R.K., Tsuchida T., Dougherty T.J. (2003). Loewen Population pharmacokinetics of the photodynamic therapy agent 2-[1-hexyloxyethyl]-2-devinyl pyropheophorbide-a in cancer patients. Am. Assoc. Cancer Res..

[19] Chen L., Zhang X., Cao Q., Wu Y., Zhang T., Tong H., Wang X., Yang J. (2019). Development and application of a physiologically based pharmacokinetic model for HPPH in rats and extrapolate to humans. Eur. J. Pharm. Sci..

[20] Gao Y.H., Zhu X.X., Zhu W., Wu D., Chen D.Y., Yan Y.J., Wu X.F., O’Shea D.F., Chen Z.L. (2020). Synthesis and evaluation of novel chlorophyll a derivatives as potent photosensitizers for photodynamic therapy. Eur. J. Med. Chem..

[21] Saenz C., Cheruku R.R., Ohulchanskyy T.Y., Joshi P., Tabaczynski W.A., Missert J.R., Chen Y., Pera P., Tracy E., Marko A. (2017). Structural and Epimeric Isomers of HPPH [3-Devinyl 3-{1-(1-hexyloxy) ethyl}pyropheophorbide-a]: Effects on Uptake and Photodynamic Therapy of Cancer. ACS Chem. Biol..

[22] Saenz C., Ethirajan M., Tracy E.C., Bowman M.-J., Cacaccio J., Ohulchanskyy T., Baumann H., Pandey R.K. (2022). Charged groups on pyropheophorbide-based photosensitizers dictate uptake by tumor cells and photodynamic therapy efficacy. J. Photochem. Photobiol. B Biol..

[23] Tan L., Shen X., He Z., Lu Y. (2022). The Role of Photodynamic Therapy in Triggering Cell Death and Facilitating Antitumor Immunology. Front. Oncol..

[24] Zenga J., Awan M., Hadi Razeghi Kondelaji M., Hansen C., Shafiee S., Frei A., Foeckler J., Kuehn R., Bruening J., Massey B. (2023). Photoactivated HPPH-Liposomal therapy for the treatment of HPV-Negative head and neck cancer. Oral Oncol..

[25] Guan L., Yang H., Cai Y., Sun L., Di P., Li W., Liu G., Tang Y. (2019). ADMET-score—A comprehensive scoring function for evaluation of chemical drug-likeness. MedChemComm.

[26] Zou Q., Fang Y., Zhao Y., Zhao H., Wang Y., Gu Y., Wu F. (2013). Synthesis and in Vitro Photocytotoxicity of Coumarin Derivatives for One- and Two-Photon Excited Photodynamic Therapy. J. Med. Chem..

[27] Zhu W., Gao Y.-H., Song C.-H., Lu Z.-B., Namulinda T., Han Y.-P., Yan Y.-J., Wang L.-X., Chen Z.-L. (2020). Synthesis and evaluation of new 5-aminolevulinic acid derivatives as prodrugs of protoporphyrin for photodynamic therapy. Photochem. Photobiol. Sci..

[28] Wang A., Zhou L., Fang K., Zhou L., Lin Y., Zhou J., Wei S. (2012). Synthesis of novel octa-cationic and non-ionic 1,2-ethanediamine substituted zinc (Ⅱ) phthalocyanines and their in vitro anti-cancer activity comparison. Eur. J. Med. Chem..

[29] Black P.N., DiRusso C.C. (2003). Transmembrane Movement of Exogenous Long-Chain Fatty Acids: Proteins, Enzymes, and Vectorial Esterification. Microbiol. Mol. Biol. Rev..

[30] Liu W., Baer M.R., Bowman M.J., Pera P., Zheng X., Morgan J., Pandey R.A., Oseroff A.R. (2007). The Tyrosine Kinase Inhibitor Imatinib Mesylate Enhances the Efficacy of Photodynamic Therapy by Inhibiting ABCG2. Clin. Cancer Res..

[31] Li Y., He G., Fu L.-H., Younis M.R., He T., Chen Y., Lin J., Li Z., Huang P. (2022). A Microneedle Patch with Self-Oxygenation and Glutathione Depletion for Repeatable Photodynamic Therapy. ACS Nano.

[32] Jamaledin R., Yiu C.K.Y., Zare E.N., Niu L.N., Vecchione R., Chen G., Gu Z., Tay F.R., Makvandi P. (2020). Advances in Antimicrobial Microneedle Patches for Combating Infections. Adv. Mater..

[33] Fu L.H., Wan Y., Qi C., He J., Li C., Yang C., Xu H., Lin J., Huang P. (2021). Nanocatalytic Theranostics with Glutathione Depletion and Enhanced Reactive Oxygen Species Generation for Efficient Cancer Therapy. Adv. Mater..

[34] Wang H., Chao Y., Liu J., Zhu W., Wang G., Xu L., Liu Z. (2018). Photosensitizer-crosslinked in-situ polymerization on catalase for tumor hypoxia modulation & enhanced photodynamic therapy. Biomaterials.

[35] Hua X.-W., Bao Y.-W., Zeng J., Wu F.-G. (2018). Ultrasmall All-In-One Nanodots Formed via Carbon Dot-Mediated and Albumin-Based Synthesis: Multimodal Imaging-Guided and Mild Laser-Enhanced Cancer Therapy. ACS Appl. Mater. Interfaces.

[36] Soo Choi H., Liu W., Misra P., Tanaka E., Zimmer J.P., Itty Ipe B., Bawendi M.G., Frangioni J.V. (2007). Renal clearance of quantum dots. Nat. Biotechnol..

[37] Cheung E.C., Vousden K.H. (2022). The role of ROS in tumour development and progression. Nat. Rev. Cancer.

[38] Tang Z., Liu Y., He M., Bu W. (2018). Chemodynamic Therapy: Tumour Microenvironment-Mediated Fenton and Fenton-like Reactions. Angew. Chem. Int. Ed..

[39] Zhang L., Wan S.-S., Li C.-X., Xu L., Cheng H., Zhang X.-Z. (2018). An Adenosine Triphosphate-Responsive Autocatalytic Fenton Nanoparticle for Tumor Ablation with Self-Supplied H2O2 and Acceleration of Fe(III)/Fe(II) Conversion. Nano Lett..

[40] Li L., Yang Z., Fan W., He L., Cui C., Zou J., Tang W., Jacobson O., Wang Z., Niu G. (2019). In Situ Polymerized Hollow Mesoporous Organosilica Biocatalysis Nanoreactor for Enhancing ROS—Mediated Anticancer Therapy. Adv. Funct. Mater..

[41] Yang W., Zhang F., Deng H., Lin L., Wang S., Kang F., Yu G., Lau J., Tian R., Zhang M. (2019). Smart Nanovesicle-Mediated Immunogenic Cell Death through Tumor Microenvironment Modulation for Effective Photodynamic Immunotherapy. ACS Nano.

[42] Yu L., Meng M., Bao Y., Zhang C., Gao B., Sa R., Luo W. (2019). miR-1301/TRIAP1 Axis Participates in Epirubicin-Mediated Anti-Proliferation and Pro-Apoptosis in Osteosarcoma. Yonsei Med. J..

[43] Liao C., Li Y., Tjong S. (2018). Graphene Nanomaterials: Synthesis, Biocompatibility, and Cytotoxicity. Int. J. Mol. Sci..

[44] Ramsey J.D., Flynn N.H. (2015). Cell-penetrating peptides transport therapeutics into cells. Pharmacol. Ther..

[45] Zhang Y.-F., Wu Y.-F., Lan T.-J., Chen Y., Su S.-H., Zhang X., Gao W., Chen J., Guo Y., Zhu J. (2021). Codelivery of Anticancer Drug and Photosensitizer by PEGylated Graphene Oxide and Cell Penetrating Peptide Enhanced Tumor-Suppressing Effect on Osteosarcoma. Front. Mol. Biosci..

[46] Hao Y., Chen Y., He X., Yu Y., Han R., Li Y., Yang C., Hu D., Qian Z. (2020). Polymeric Nanoparticles with ROS—Responsive Prodrug and Platinum Nanozyme for Enhanced Chemophotodynamic Therapy of Colon Cancer. Adv. Sci..

[47] Jiang M., Mu J., Jacobson O., Wang Z., He L., Zhang F., Yang W., Lin Q., Zhou Z., Ma Y. (2020). Reactive Oxygen Species Activatable Heterodimeric Prodrug as Tumor-Selective Nanotheranostics. ACS Nano.

[48] Hao Y., Chen Y., He X., Han R., Yang C., Liu T., Yang Y., Liu Q., Qian Z. (2023). RGD peptide modified platinum nanozyme Co-loaded glutathione-responsive prodrug nanoparticles for enhanced chemo-photodynamic bladder cancer therapy. Biomaterials.

[49] Liu H., Mei C., Deng X., Lin W., He L., Chen T. (2021). Rapid visualizing and pathological grading of bladder tumor tissues by simple nanodiagnostics. Biomaterials.

[50] Wang S., Yu G., Yang W., Wang Z., Jacobson O., Tian R., Deng H., Lin L., Chen X. (2021). Photodynamic—Chemodynamic Cascade Reactions for Efficient Drug Delivery and Enhanced Combination Therapy. Adv. Sci..

[51] Wang Y., Xie Y., Li J., Peng Z.-H., Sheinin Y., Zhou J., Oupický D. (2017). Tumor-Penetrating Nanoparticles for Enhanced Anticancer Activity of Combined Photodynamic and Hypoxia-Activated Therapy. ACS Nano.

[52] Yin L., Duan J.-J., Bian X.-W., Yu S.-C. (2020). Triple-negative breast cancer molecular subtyping and treatment progress. Breast Cancer Res..

[53] Zheng Z., Shao N., Weng H., Li W., Zhang J., Zhang L., Yang L., Ye S. (2014). Correlation between epidermal growth factor receptor and tumor stem cell markers CD44/CD24 and their relationship with prognosis in breast invasive ductal carcinoma. Med. Oncol..

[54] Yang R., Lu M., Ming L., Chen Y., Cheng K., Zhou J., Jiang S., Lin Z., Chen D. (2020). ^89^Zr-Labeled Multifunctional Liposomes Conjugate Chitosan for PET-Trackable Triple-Negative Breast Cancer Stem Cell Targeted Therapy. Int. J. Nanomed..

[55] Ding Y., Yang R., Yu W., Hu C., Zhang Z., Liu D., An Y., Wang X., He C., Liu P. (2021). Chitosan oligosaccharide decorated liposomes combined with TH302 for photodynamic therapy in triple negative breast cancer. J. Nanobiotechnology.

[56] Ogbonna S.J., Hazama H., Awazu K. (2021). Mass Spectrometric Analysis of the Photobleaching of Protoporphyrin IX Used in Photodynamic Diagnosis and Therapy of Cancer. Photochem. Photobiol..

[57] Haedicke K., Kozlova D., Gräfe S., Teichgräber U., Epple M., Hilger I. (2015). Multifunctional calcium phosphate nanoparticles for combining near-infrared fluorescence imaging and photodynamic therapy. Acta Biomater..

[58] James N.S., Joshi P., Ohulchanskyy T.Y., Chen Y., Tabaczynski W., Durrani F., Shibata M., Pandey R.K. (2016). Photosensitizer (PS)-cyanine dye (CD) conjugates: Impact of the linkers joining the PS and CD moieties and their orientation in tumor-uptake and photodynamic therapy (PDT). Eur. J. Med. Chem..

[59] James N.S., Ohulchanskyy T.Y., Chen Y., Joshi P., Zheng X., Goswami L.N., Pandey R.K. (2013). Comparative Tumor Imaging and PDT Efficacy of HPPH Conjugated in the Mono- and Di-Forms to Various Polymethine Cyanine Dyes: Part 2. Theranostics.

[60] Carr J.A., Valdez T.A., Bruns O.T., Bawendi M.G. (2016). Using the shortwave infrared to image middle ear pathologies. Proc. Natl. Acad. Sci. USA.

[61] Chepurna O.M., Yakovliev A., Ziniuk R., Nikolaeva O.A., Levchenko S.M., Xu H., Losytskyy M.Y., Bricks J.L., Slominskii Y.L., Vretik L.O. (2020). Core–shell polymeric nanoparticles co-loaded with photosensitizer and organic dye for photodynamic therapy guided by fluorescence imaging in near and short-wave infrared spectral regions. J. Nanobiotechnology.

[62] Zhang S., Cheruku R.R., Dukh M., Tabaczynski W., Patel N.J., White W.H., Missert J.R., Spernyak J.A., Pandey R.K. (2020). The Structures of Gd(III) Chelates Conjugated at the Periphery of 3-(1’-Hexyloxy)ethyl-3-devinylpyropheophorbide-a (HPPH) Have a Significant Impact on the Imaging and Therapy of Cancer. ChemMedChem.

[63] Cheruku R.R., Turowski S.G., Durrani F.A., Tabaczynski W.A., Cacaccio J., Missert J.R., Curtin L., Sexton S., Alberico R., Hendler C.M. (2022). Tumor-Avid 3-(1’-Hexyloxy)ethyl-3-devinylpyrpyropheophorbide-a (HPPH)-3Gd(III)tetraxetan (DOTA) Conjugate Defines Primary Tumors and Metastases. J. Med. Chem..

[64] Ardeshirpour Y., Chernomordik V., Capala J., Hassan M., Zielinsky R., Griffiths G., Gandjbakhche A. (2011). Using in vivo fluorescence imaging in personalized cancer diagnosis and therapy, an image and treat paradigm. Technol. Cancer Res. Treat..

[65] Borah B.M., Cacaccio J., Watson R., Pandey R.K. (2019). Phototriggered Release of Tumor-Imaging and Therapy Agents from Lyophilized Multifunctional Polyacrylamide Nanoparticles. ACS Appl. Bio Mater..

[66] Weżgowiec J., Kulbacka J., Saczko J., Rossowska J., Chodaczek G., Kotulska M. (2018). Biological effects in photodynamic treatment combined with electropermeabilization in wild and drug resistant breast cancer cells. Bioelectrochemistry.

[67] Algorri J.F., Ochoa M., Roldán-Varona P., Rodríguez-Cobo L., López-Higuera J.M. (2021). Light Technology for Efficient and Effective Photodynamic Therapy: A Critical Review. Cancers.

[68] Tracy E.C., Bowman M.-J., Pandey R.K., Baumann H. (2022). Tumor cell-specific retention of photosensitizers determines the outcome of photodynamic therapy for head and neck cancer. J. Photochem. Photobiol. B Biol..

